# Quercetin induces cell apoptosis of myeloma and displays a synergistic effect with dexamethasone *in vitro* and *in vivo* xenograft models

**DOI:** 10.18632/oncotarget.9993

**Published:** 2016-06-14

**Authors:** Donghua He, Xing Guo, Enfan Zhang, Fuming Zi, Jing Chen, Qingxiao Chen, Xuanru Lin, Li Yang, Yi Li, Wenjun Wu, Yang Yang, Jingsong He, Zhen Cai

**Affiliations:** ^1^ Bone Marrow Transplantation Center, The First Affiliated Hospital, School of Medicine, Zhejiang University, Hangzhou, Zhejiang, China; ^2^ Department of Hematology, The Second Affiliated Hospital of Nanchang University, Nanchang, Jiangxi, China

**Keywords:** apoptosis, dexamethasone, multiple myeloma, quercetin

## Abstract

Quercetin, a kind of dietary flavonoid, has shown its anticancer activity in many kinds of cancers including hematological malignancies (acute myelogenous leukemia, chronic myelogenous leukemia, chronic lymphocytic leukemia, and MM) *in vitro* and *in vivo*. However, its effects on MM need further investigation. In this study, MM cell lines were treated with quercetin alone or in combination with dexamethasone. In order to observe the effects *in vivo*, a xenograft model of human myeloma was established. Quercetin inhibited proliferation of MM cells (RPMI8226, ARP-1, and MM.1R) by inducing cell cycle arrest in the G2/M phase and apoptosis. Western blot showed that quercetin downregulated c-myc expression and upregulated p21 expression. Quercetin also activated caspase-3, caspase-9, and poly(ADP-ribose)polymerase 1. Caspase inhibitors partially blocked apoptosis induced by quercetin. Furthermore, quercetin combined with dexamethasone significantly increased MM cell apoptosis. In vivo xenograft models, quercetin obviously inhibited tumor growth. Caspase-3 was activated to a greater extent when quercetin was combined with dexamethasone. In conclusion, quercetin alone or in combination with dexamethasone may be an effective therapy for MM.

## INTRODUCTION

Multiple myeloma (MM) is a hematological malignancy with plasma cell proliferative disorder. Its incidence has increased recently. The major clinical features of MM include increased blood calcium level, renal insufficiency, anemia, and bone lesions (CRAB). During the last few years, the survival of MM patients improved greatly with the use of novel agents (bortezomib, carfilzomib, thalidomide, lenalidomide, and others) and autologous stem cell transplantation [1, 2]. However, the majority of MM patients eventually relapsed [3–5]. As a result, MM is still an incurable disease and hence novel therapeutic agents need to be developed for MM patients.

Quercetin (3,5,7,3′,4′-pentahydroxyflavone) is a kind of flavonoid that exists abundantly in Chinese diets and herbs. In many studies, quercetin has displayed antioxidative, anti-inflammatory, and antitumor effects [6–9]. The anticancer activity of quercetin has been shown in many kinds of cancers including solid tumors [10–12] and hematological malignancies by inducing apoptosis, autophagy, and cell cycle arrest, and in downregulating glycolytic metabolism [13–16]. Furthermore, some studies found that quercetin could enhance tumor cell sensitivity to vincristine [17], doxorubicin [8, 15], and 5-fluorouracil [18], and reverse multidrug resistance of cancers [17, 19–22]. A previous study found that quercetin suppressed the proliferation of MM cells by downregulating the expression of IQ motif-containing GTPase activating protein 1 and the activation of extracellular signal-regulated kinase [23]. The present study aimed to explore an alternative mechanism of the inhibition of MM cell proliferation by quercetin and investigate the effect of quercetin combined with dexamethasone on MM.

## RESULTS

### Quercetin inhibited proliferation of MM cell lines

**Figure 1:**
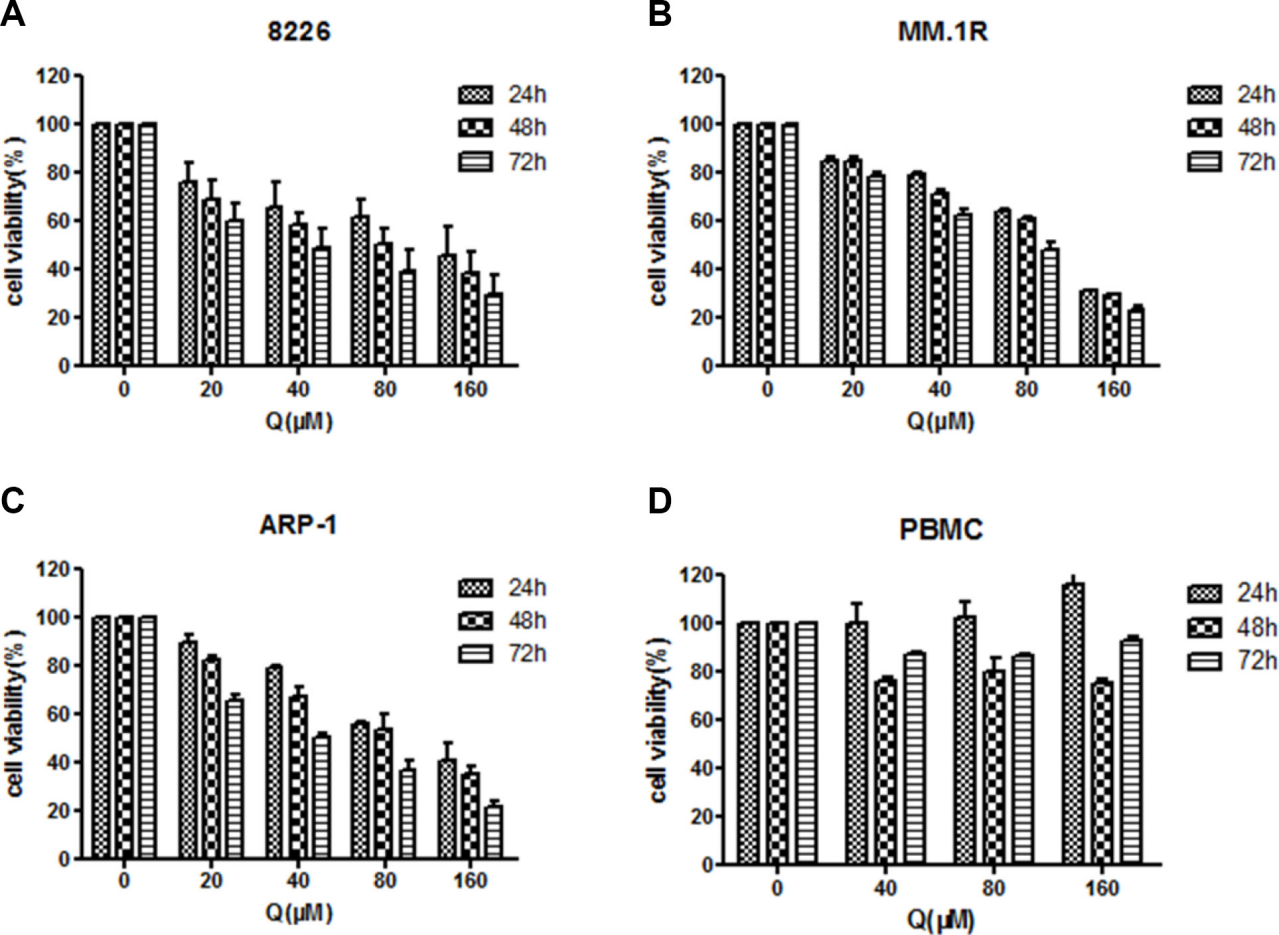
Effect of quercetin on myeloma cell viability measured by MTT assay (**A**–**C**) Cell proliferation in three myeloma cell lines at 24, 48, and 72 h after treatment with different doses of quercetin. RPMI8226, MM.1R, and ARP-1 cells were cultured in 96-well plates in the presence of 0–160 μM quercetin. (**D**) Peripheral blood mononuclear cells isolated from healthy individuals (*n* = 3) tested at 24, 48, and 72 h after treatment with different doses of quercetin. The data were obtained from three independent experiments and presented as mean ± SD; Q, quercetin.

The study examined the effect of quercetin on different MM cell lines by treating MM cells with different doses of quercetin for 24, 48, and 72 h. MTT showed that quercetin inhibited MM cell proliferation in a dose- and time-dependent manner, as shown in Figure 1A–1C. However, the proliferation of peripheral blood mononuclear cells isolated from healthy individuals (*n* = 3) was not significantly inhibited, suggesting that quercetin had little cytotoxic effect on normal mononuclear cells, as shown in Figure 1D.

### Quercetin caused MM cell cycle arrest

**Figure 2:**
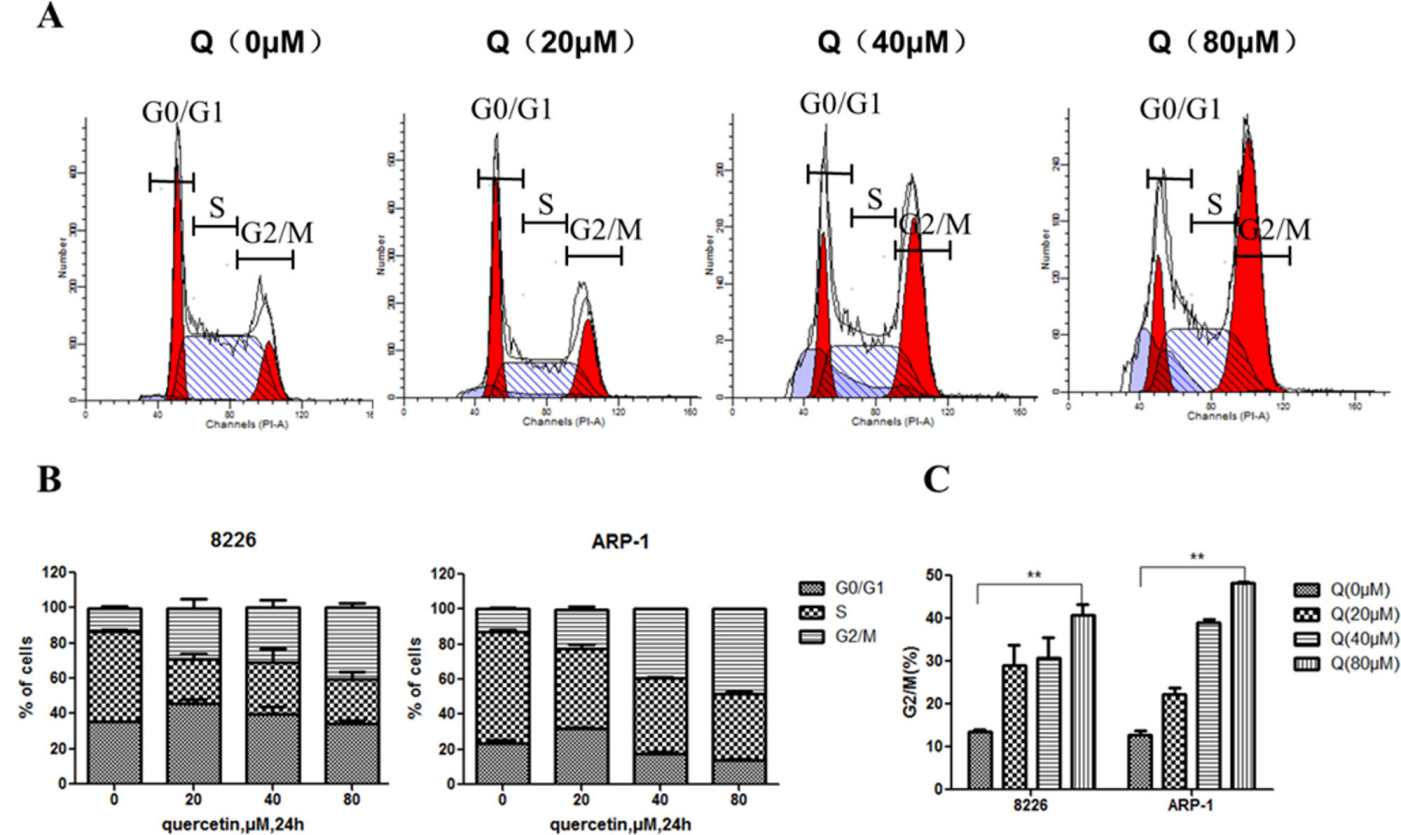
Cell cycle of MM cells treated with quercetin (**A**) Representative results showing the percentage of cells in G0/G1, S, and G2/M phases in ARP-1 with quercetin (0, 20, 40, and 80 μM) treatment for 24 h, as detected by flow cytometry. (**B** and **C**) Histograms showing the percentage of cells in G0/G1, S, and G2/M phases in three independent experiments; Q, quercetin, **P < 0.01.

To investigate the antiproliferative mechanism of quercetin in MM cells, the cells were treated with different doses of quercetin for 24 h, and the cell cycle was analyzed using flow cytometry. As shown in Figure 2A and 2B, quercetin treatment resulted in cell cycle arrest in the G2/M phase compared with the control.

### Quercetin-induced MM cell line and primary MM cell apoptosis, which could be partially blocked by caspase inhibitor

**Figure 3:**
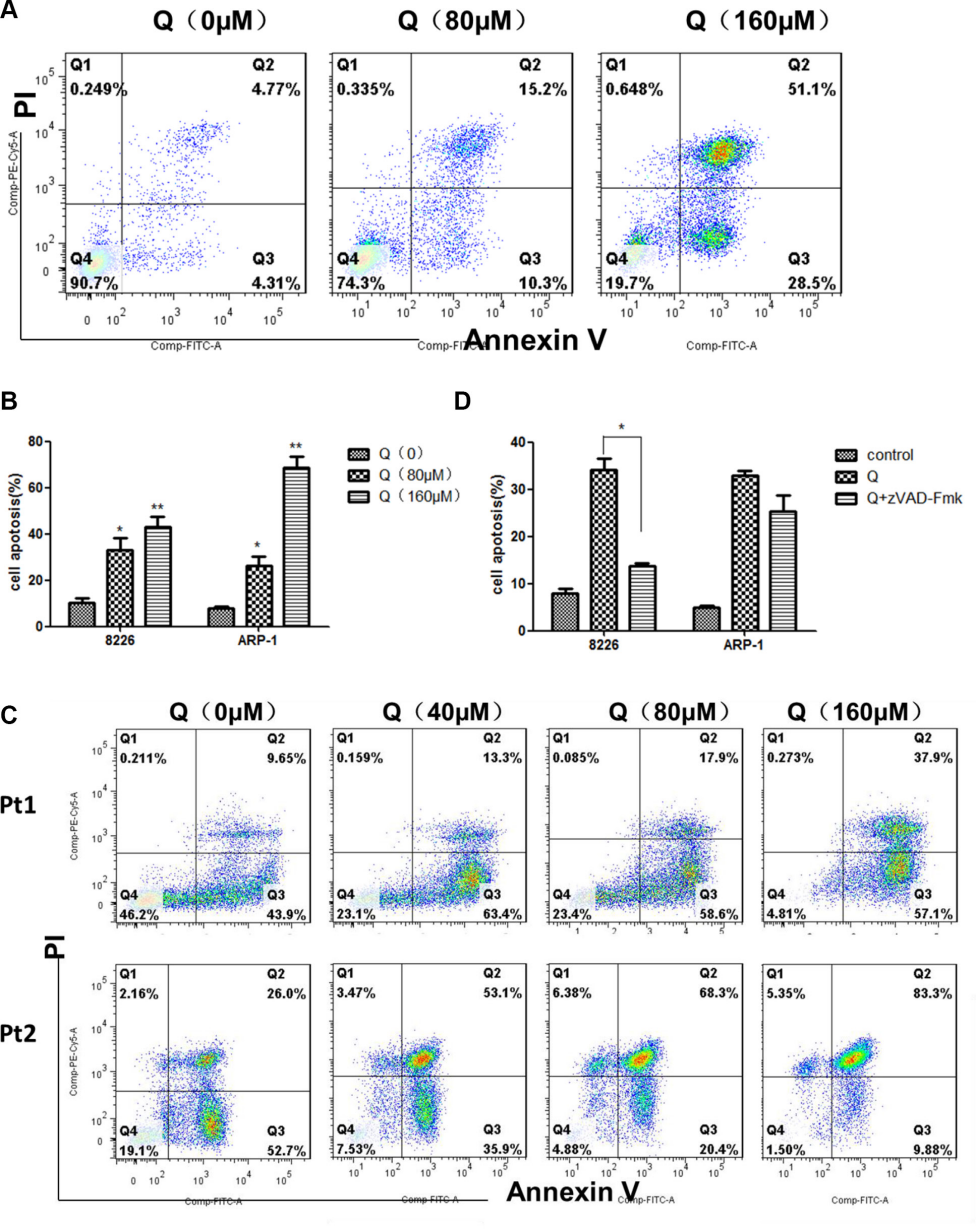
Effect of quercetin on myeloma cell apoptosis (**A**) Representative results showing the percentage of ARP-1 in apoptosis after treatment with quercetin (0, 80, and 160 μM) for 24 h, as detected by flow cytometry. AnnexinV-positive cells were considered as apoptotic cells. (**B**) Histograms showing the percentage of cells in apoptosis. RPMI8226 and ARP-1 were treated with quercetin in three independent experiments. (**C**) Representative results showing the percentage of primary MM cells in apoptosis after treatment with quercetin (0, 40, 80, and 160 μM) for 24 h, as detected by flow cytometry. (**D**) MM cells were treated with quercetin at 160μM in the presence of Z-VAD-FMK (50 μM) for 24 h, and then collected for analyzing apoptosis using flow cytometry. *P < 0.05; **P < 0.01 (quercetin vs medium).

To evaluate the proapoptotic effect of quercetin, MM cell lines and primary MM cells were treated with quercetin for 24 h and apoptosis was measured by Annexin V/PI staining. Annexin V-positive cells were considered as apoptotic cells. As shown in Figure 3A and 3B, quercetin had a significant proapoptotic effect on MM cells. As shown in Figure 3C, quercetin also induced primary MM cell apoptosis. To determine whether apoptosis induced by quercetin was only through the caspase pathway, MM cells were treated with quercetin in the presence of Z-VAD-FMK, a pan-caspase inhibitor, and it was found that the pan-caspase inhibitor Z-VAD-FMK could block the proapoptotic effect of quercetin on RPMI8226 and ARP-1, but the blocking effect on ARP-1 was not statistically significant, as shown in Figure 3D.

### Quercetin-induced MM cell apoptosis and cell cycle arrest were confirmed by Western blot

**Figure 4:**
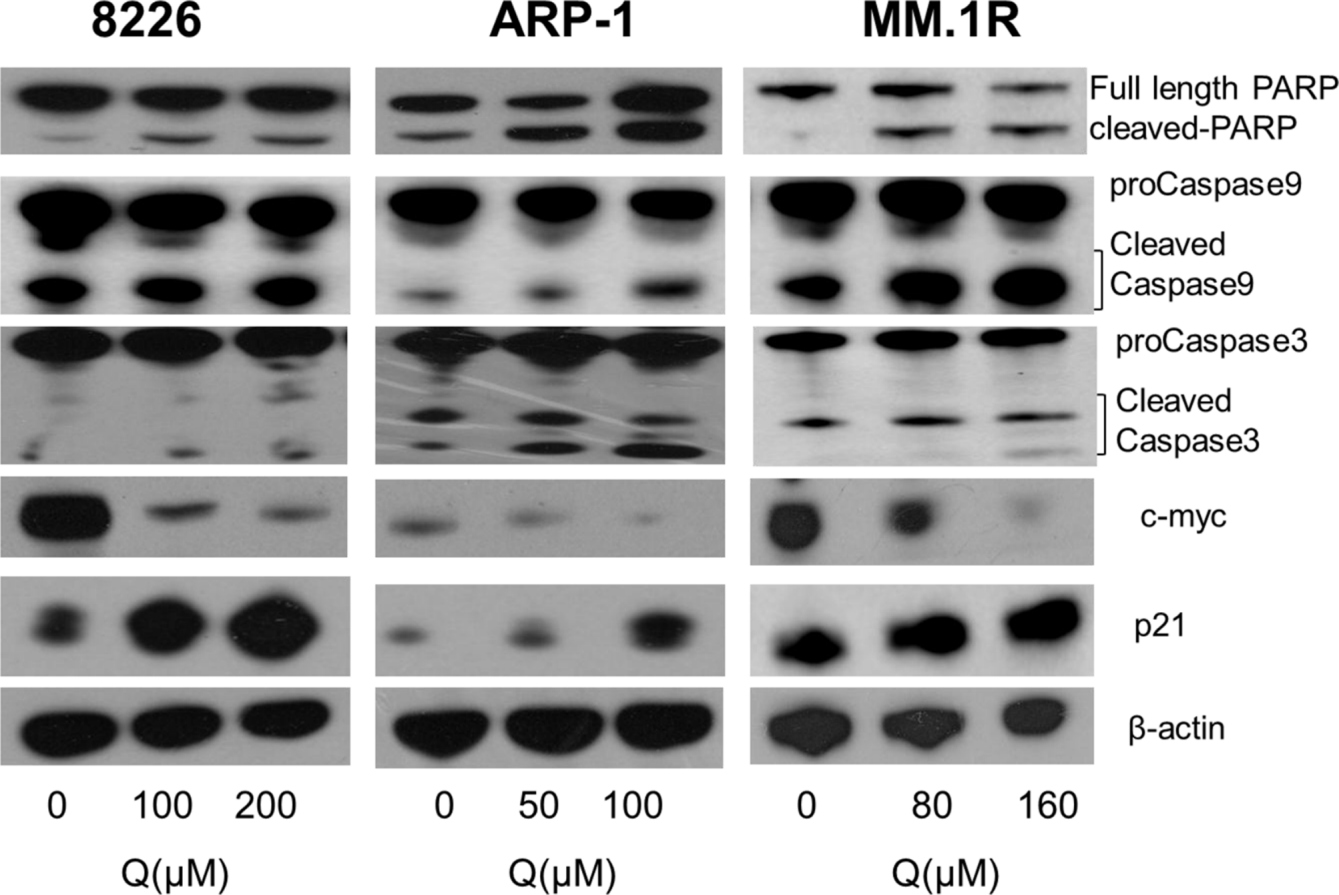
Changes in apoptosis- and cell cycle–related proteins caspase-3, caspase-9, PARP1, c-*myc*, and p21 were shown after quercetin treatment, as detected by Western blot; Q, quercetin.

To confirm the aforementioned results, the changes in apoptosis-related and cell cycle–related proteins were further tested by Western blot. As shown in Figure 4, caspase-3, caspase-9, and PARP-1 were activated after treatment with quercetin (0–200 μM), and the expression of cell cycle proteins such as p21 was significantly increased. However, the expression of c-*myc*, an important oncogene for MM cells, was markedly downregulated.

### Quercetin displayed a synergistic inhibition effect with dexamethasone in *in vitro* and *in vivo* xenograft models

**Figure 5:**
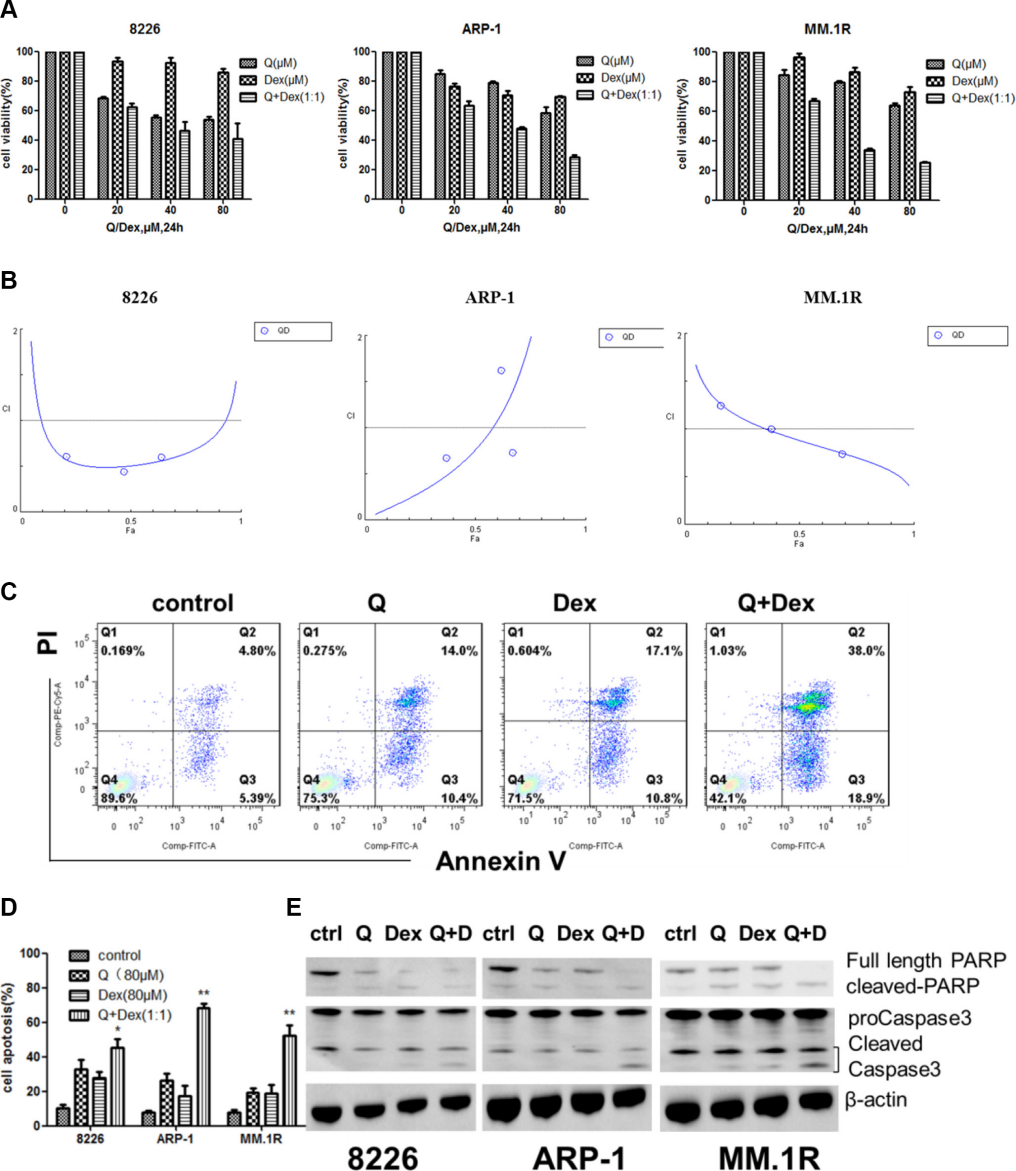
Combined effect of quercetin and dexamethasone (**A**) Cell viability of RPMI8226, ARP-1, and MM.1R cells exposed to quercetin (0, 20, 40, and 80 μM), dexamethasone (0, 20, 40, and 80μM), or the combination (Q:Dex = 1:1) was detected using MTT. (**B**) The Fa-CI plot showed the combination index (CI value) for each fractional effect. The curves were generated using CompuSyn software. The results showed that quercetin had a synergistic effect with dexamethasone (CI < 1). (**C**) The synergistic effect occurred mainly through apoptosis. MM.1R cells were exposed to quercetin (80 μM), dexamethasone (80 μM), or the combination for 24 h. (**D**) Histograms showed the percentage of apoptotic cells of RPMI8226, ARP-1, and MM.1R treated with quercetin (80 μM), dexamethasone (80 μM), or the combination for 24 h detected using flow cytometry. (**E**) Western blot was performed to confirm the combination effect. Q, quercetin; Dex, dexamethasone; Q + Dex, quercetin + dexamethasone, **P < 0.05, **P < 0.01.

**Figure 6:**
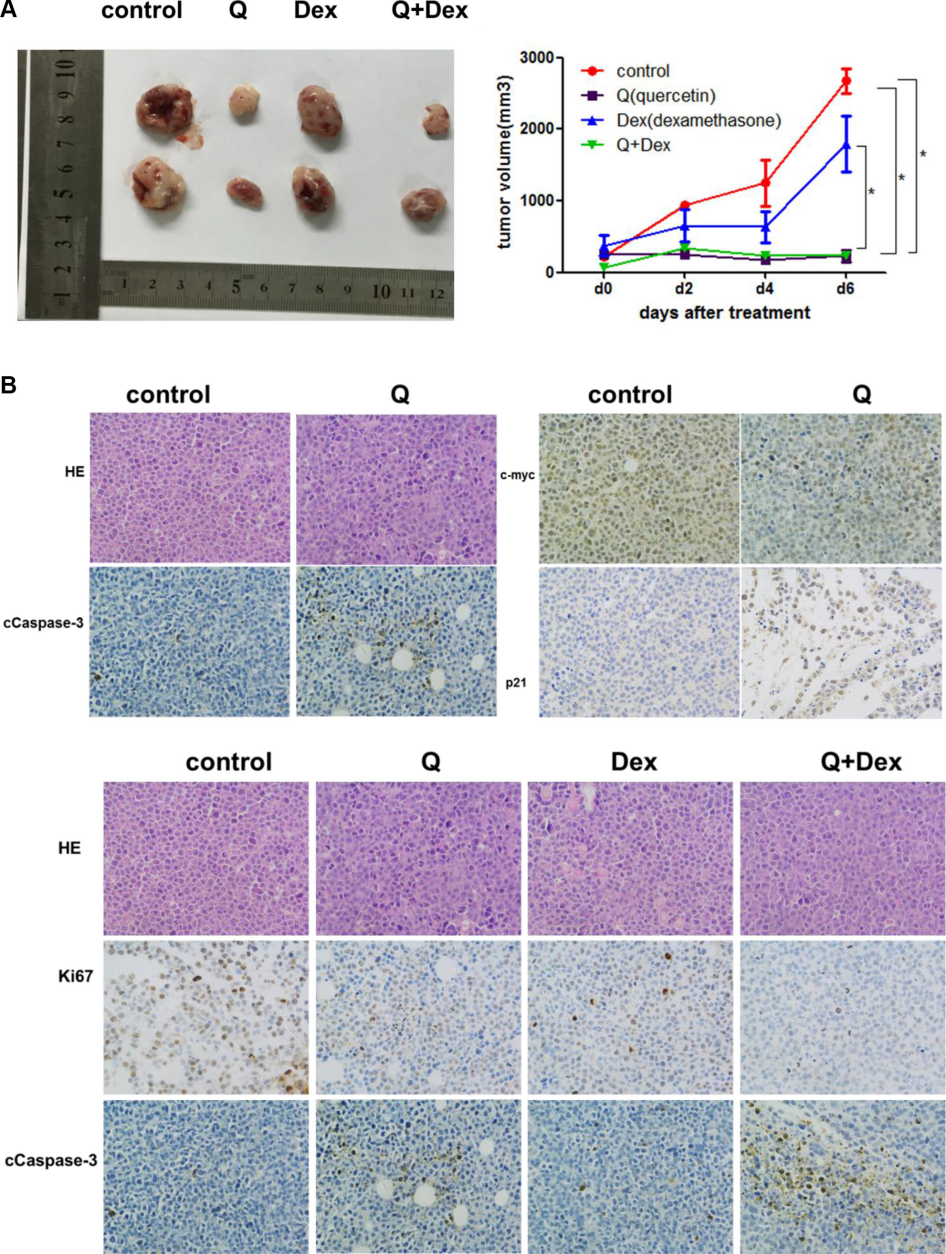
*In vivo* effects of quercetin or quercetin + dexamethasone treatment on established myeloma in NOD–SCID mice (**A**) Tumor volume in different treatment groups (three to four mice per group; *P < 0.05). (**B**) Immunohistochemistry analysis with anti-cleaved caspase-3, anti-c-*myc*, and anti-p21 antibodies (magnification: ×200), *P < 0.05.

To test whether quercetin has a synergistic, an additive, or an antagonistic effect when used in combination with dexamethasone, MM cells RPMI8226, ARP-1, and MM.1R were treated with quercetin and dexamethasone. The effects were analyzed using the CompuSyn software. First, the combination effect of quercetin and dexamethasone was tested, as shown in Figure 5A. RPMI8226, ARP-1, and MM.1R were treated with different doses of quercetin (0, 20, 40, and 80 μM) and similar doses of dexamethasone (0, 20, 40, and 80 μM) for 24 h, and the cell viability was measured using MTT. As shown in Figure 5B, quercetin had a synergistic effect with dexamethasone (CI < 1). To further examine the mechanism of the combined effect of quercetin and dexamethasone, MM cells were exposed to quercetin with or without dexamethasone, and the apoptotic cells were detected using Annexin V/PI staining with flow cytometry. As shown in Figure 5C and 5D, compared with the single drug treatment group, the percentage of apoptosis significantly increased in the combination treatment group. The results were further verified by Western blot, as shown in Figure 5E. To test the antimyeloma activity of quercetin and its synergistic effect with dexamethasone *in vivo*, a human NOD–SCID–MM mouse model using ARP-1 cells was established. The mice that were exposed to quercetin had a lower tumor burden compared with the control group. However the tumor burden in the group treated with quercetin and dexamethasone combination was not different from the group treated with quercetin alone, as shown in Figure 6A. Immunohistochemical analysis showed that quercetin inhibited the proliferation of MM cells, activated caspase-3 and p21, and downregulated c-myc expression. Importantly, quercetin combined with dexamethasone activated caspase-3 to a greater extent than quercetin or dexamethasone alone, as shown in Figure 6B.

## DISCUSSION

Previous studies have shown that the flavonoids such as quercetin have anticancer effects in both solid tumors and hematological malignancies [6, 10, 13, 15, 24, 25]. A study by Yongyong et al. in 2014 has demonstrated that quercetin suppresses the proliferation of MM cells by downregulating the expression of IQ motif-containing GTPase activating protein 1 and activating extracellular signal-regulated kinase [23]. Our study found that quercetin induced cell cycle arrest and apoptosis of myeloma cells by using *in vitro* and *in vivo* models. More importantly, it was found that quercetin displayed a synergistic antimyeloma effect with dexamethasone.

The present study investigated the proapoptotic activity of quercetin by activating caspase-3 and caspase-9, which was consistent with the studies on leukemia cells [16]. It was also found that the pan-caspase inhibitor Z-VAD-FMK could block the proapoptotic effect of quercetin on RPMI8226 and ARP-1, but the blocking effect on ARP-1 was not statistically significant, which suggested that the proapoptotic effect of quercetin was partly through the caspase pathway. However, other mechanisms of cell apoptosis induced by quercetin existed, such as autophagy or necrosis [10], which might explain that the proapoptotic effect of quercetin on ARP-1 could not be totally reversed by Z-VAD-FMK. These alternative mechanisms need further investigation.

Previous study showed that quercetin induced apoptosis by inhibiting proteasome activition, causing G2/M phase arrest and increasing p21 expression [26–29]. Our data also showed that quercetin induced MM cell cycle arrest in the G2/M phase. Western blot demonstrated an increase in p21 expression, which suggested that quercetin treatment could lead to DNA damage in MM cells, and before the repair of the damage, the cells did not initiate mitosis. The myc protein encoded by *myc* gene was involved in regulating many important proteins associated with cell protein biosynthesis, energy metabolism, proliferation, and apoptosis that contribute to the genesis of many human cancers [30–32]. Gene expression studies on the role of c-*myc* in multiple myeloma indicated that c-*myc* could be detected in approximately 70% of primary myeloma clones in contrast to cells from the premalignant condition, monoclonal gammopathy of undetermined significance [33]; c-*myc* has also been shown to be important for the survival of MM cell lines because the downregulation of c-*myc* by RNA interference induces apoptosis in some myeloma cell lines [34–36]. The present study demonstrated that c-*myc* expression was downregulated obviously and p21 expression increased after treatment with quercetin, which indicated that quercetin might induce MM cell cycle arrest in the G2/M phase and apoptosis through c-*myc* downregulation. However, the mechanism underlying the influence of quercetin on c-*myc* expression remains unclear.

A study by Feng-Ting Liu found that chronic lymphocytic leukemia (CLL) cells were sensitive *in vitro* to bortezomib and dietary flavonoids, quercetin and myricetin, inhibited bortezomib-induced apoptosis of primary CLL and malignant B-cell lines in a dose-dependent manner. It was further found that this inhibitory effect was associated with chemical reactions between quercetin and the boronic acid group, −RB(OH)2, in bortezomib [26]. Bortezomib resistance occurred in many MM patients [4, 5, 37]. Also, being a proteasome inhibitor [27], whether quercetin had the same antimyeloma effect in MM cells that were resistant to bortezomib is still unclear and needs to be investigated further. The first-line therapies for newly diagnosed MM always include dexamethasone, such as PD, PCD, PAD, and PTD [1]. Dexamethasone alone also could inhibit MM cell proliferation and induce apoptosis though the effect was limited. The present study showed a significantly synergistic effect of quercetin with dexamethasone through apoptosis. Thus, the data suggest that the combined treatment of quercetin with dexamethasone may be an effective therapy for MM patients.

The *in vivo* experiments demonstrated that the mice exposed to quercetin had a lower tumor burden compared with the control group but the tumor burden in the group treated with the quercetin and dexamethasone combination was not different from the group treated with quercetin alone. However, the immunohistochemical analysis showed that quercetin combined with dexamethasone activated caspase-3 to a greater extent compared with quercetin or dexamethasone alone. Maybe the dose of quercetin was high enough to inhibite the proliferation of tumor cells alone. Therefore, the synergistic inhibition effect with dexamethasone was not achieved phenotypically.

In conclusion, the present study demonstrated that quercetin has antimyeloma activity both *in vitro* and *in vivo*. Quercetin alone or in combination with dexamethasone may be an effective therapy for MM.

## MATERIALS AND METHODS

### Human myeloma cell lines, primary myeloma cells, and healthy peripheral blood mononuclear cells

Human MM cell lines RPMI8226, MM.1R, and ARP-1 were kindly provided by Dr. Qing Yi (Department of Cancer Biology, Lerner Research Institute, Cleveland Clinic, Cleveland, OH, USA). Primary CD138+ cells of bone marrow from MM patients and peripheral blood mononuclear cells from healthy individuals were obtained after informed consent from donors and approval by the Ethics Committee of the First Affiliated Hospital, Zhejiang University School of Medicine. CD138+ cells were collected using positive selection with CD138 microbeads (Miltenyi Biotech, CA, USA).

### Reagents and antibodies

Quercetin, dexamethasone, 3-(4,5-dimethylthiazol-2-yl)-2,5 diphenyltetrazolium bromide (MTT), dimethyl sulfoxide (DMSO), propidium iodide (PI), and ribonuclease A were all purchased from Sigma(St. Louis, MO, USA) Z-VAD-FMK was purchased from R&D systems (USA). Annexin V Apoptosis Detection Kit FITC/PI was obtained from eBioscience (CA, USA). Primary antibodies against caspase-3, caspase-8, caspase-9, poly(ADP-ribose)polymerase 1 (PARP-1), and c-myc were procured from Cell Signaling Technology (MA, USA). Primary antibody against p21 was obtained from Abcam (Cambridge, UK). Primary antibody against β-actin was obtained from Sigma-Aldrich. Horseradish peroxidase (HRP)–conjugated antirabbit and antimouse antibodies were procured from Jackson Immuno Research Laboratories (PA, USA).

### Cell culture

RPMI8226, ARP-1, MM.1R, and primary cells were cultured in RPMI 1640 medium (Thermo Scientific, Hyclone) supplemented with 10% fetal bovine serum (Thermo Fisher Scientific, Gibco), and 1% L-glutamine at 37°C in a humidified atmosphere and 5% CO_2_.

### Cell proliferation assays and synergy analysis

The MTT assay was used to detect MM cell proliferation. MM cells (1–2 × 10^4^/well) were plated in 96-well plates and treated with or without quercetin or dexamethasone (0–160 μM) at 37°C in a humidified atmosphere and 5% CO_2_. After 24, 48, and 72 h, the cells were treated with 20 μL of MTT solution (5 mg/mL) and incubated at 37°C for another 4 h. Then, absorbance was measured at 570 nm using a microplate reader (Bio-Rad, Model 680); cell viability (%) = OD value of test sample/OD value of control × 100%. To determine the effect of combination treatment (quercetin plus dexamethasone) at 24 h, CompuSyn software was used. For this synergy analysis, quercetin was mixed with dexamethasone in a constant ratio. The combination effect was quantified based on a combination index (CI) to assess synergism (CI < 1), additive effect (CI = 1), and antagonism (CI > 1).

### Flow cytometry: Apoptosis and cell cycle

To detect apoptotic cells, 1 × 10^5^/mL MM cells (RPMI8226, ARP-1, and MM.1R) were plated in 12-well plates for 24 h with quercetin alone or quercetin combined with dexamethasone at a constant ratio or Z-VAD-FMK. Then, the cells were harvested, washed twice with phosphate-buffered saline (PBS), resuspended in 200–300 μL of staining buffer, and stained with Annexin V–FITC/PI according to the manufacturer's instructions. The cells were detected using flow cytometry, and the data were analyzed using FlowJo7.6.1.

RPMI8226 and ARP-1 cells (2 × 10^5^/well) were cultured with different doses of quercetin (0, 20, 40, and 80 μM) in six-well plates for 48 h. The cells were washed twice with PBS and then permeabilized with precooled 75% ethanol at 4°C overnight. The next day, the cells were washed twice with PBS, treated with 0.01% RNase A for 30 min at 37°C, and then incubated with 0.5% PI. The cells were detected using flow cytometry (BD Biosciences, CA, USA), and the data were analyzed using ModFit software (version 3.2, Verity Software House).

### Western blot analysis

MM cell lines treated with quercetin alone or in combination with dexamethasone were washed twice with PBS and extracted with RIPA. The supernatants were collected for Western blot. The proteins (20–40 μg) were separated by 8%–12% sodium dodecyl sulfate–polyacrylamide gel electrophoresis and transferred to polyvinylidene difluoride membranes (Merck Millipore, Germany). The membranes were blocked with 5% nonfat milk for 1–2 h and then incubated with specific primary antibodies overnight at 4°C. The next day, the membranes were washed using Tris-buffered saline with Tween 20 (TBS-T) and then incubated with horseradish peroxidase (HRP)–conjugated antirabbit or antimouse antibodies at room temperature for 1 h. The membranes were washed with TBS-T again, and the image was detected using an x-ray film with an enhanced chemiluminescence detection kit for HRP (Biological Industries, Israel, Beit Haemek Ltd.).

### Human tumor xenografts in NOD–SCID mice

To observe the antimyeloma effect of quercetin alone or in combination with dexamethasone *in vivo*, a xenograft model of human myeloma was established. Four-week-old male NOD–SCID mice were purchased from Vital River Laboratory Animal Technology Co. Ltd. (Beijing, China), and then the NOD–SCID mice were injected with 1 × 10*7* ARP-1 cells subcutaneously in the right flank. After about 1 week, when the established tumors reached about 100–130 mm*3*, the mice were randomly divided into four groups and started to receive an intraperitoneal injection of vehicle or quercetin alone [150 mg/(kg·× day), for a 7-day continuum] or dexamethasone alone [15 mg/(kg·× day), for a 7-day continuum] or a combination of quercetin [150 mg/(kg·× day), for a 7-day continuum] and dexamethasone [15 mg/(kg·× day), for a 7-day continuum]. The tumor diameter was measured every 3 days using calipers, and the tumor volume was calculated as 4π/3 × (*a*/2)2 × *b*/2, where *a* is the width and *b* is the length. All experiments followed the procedures and protocols of the Animal Ethics Committee of the First Affiliated Hospital, Zhejiang University School of Medicine.

### Immunohistochemistry

Tumor tissue samples derived from the NOD–SCID mice were fixed in 4% paraformaldehyde, embedded in paraffin, and sectioned (5 μm). For immunohistochemical staining, the steps were as described earlier [38].

### Statistical analysis

All results were presented as mean ± standard deviation (SD). A two-tailed Student's *t* test was used to determine the statistical differences between two groups, and a one-way analysis of variance was used to estimate the differences between three or more groups. All *P* values less than 0.05 were considered statistically significant. All analyses were performed using GraphPad Prism 5.0 (GraphPad Software, CA, USA).
